# Codesigning a patient support portal with health professionals and men with prostate cancer: An action research study

**DOI:** 10.1111/hex.13444

**Published:** 2022-04-11

**Authors:** Benjamin Shemesh, Jacinta Opie, Ellie Tsiamis, Darshini Ayton, Prassannah Satasivam, Paula Wilton, Karla Gough, Katrina Lewis, Colin O'Brien, Max Shub, Amanda Pomery, Christopher Mac Manus, Jeremy Millar, Susan Evans

**Affiliations:** ^1^ School of Public Health and Preventive Medicine Monash University Melbourne Victoria Australia; ^2^ Department of Surgery, Northern Health The University of Melbourne Melbourne Victoria Australia; ^3^ The Victorian Agency for Health Information (VAHI) Melbourne Victoria Australia; ^4^ Health Services Research Peter MacCallum Cancer Centre Melbourne Victoria Australia; ^5^ Patient Experience and Consumer Participation, Alfred Health Melbourne Victoria Australia; ^6^ Movember Melbourne Victoria Australia; ^7^ Helix Monash University Melbourne Victoria Australia; ^8^ Radiation Oncology Alfred Health Melbourne Victoria Australia; ^9^ Cancer Council Victoria Melbourne Victoria Australia

**Keywords:** action research study, codesign, patient‐reported outcomes, prostate cancer, support portal

## Abstract

**Introduction:**

The supportive care needs of men with prostate cancer (PCa) have been well documented, but little is known about how an online portal may address these. This study sought to determine priority issues facing men with PCa, barriers and enablers to accessing care and whether health professionals (HPs) and men would support the inclusion of a patient‐reported outcome (PRO) comparator tool.

**Methods:**

We conducted four online focus groups with HPs recruited from healthcare services in Victoria, followed by seven online codesign workshops with men with PCa, recruited through the Victorian Prostate Cancer Outcomes Registry, Prostate Cancer Foundation Australia and the Cancer Council Victoria. Men were eligible to participate if they had lived experience of PCa and access to the internet. We analysed focus groups thematically. Workshops were analysed using descriptive‐content analysis.

**Results:**

HPs (*n* = 39) highlighted that men had shifting priorities over time, but noted the importance of providing information to men in lay terms to assist in treatment decision‐making and side‐effect management. HPs identified key enablers to men accessing support services such as practice nurses, partners and having men share their stories with each other. HPs raised financial, cultural, geographic and emotional barriers to accessing supportive care. Inclusion of a PRO comparator tool received mixed support from HPs, with 41% (*n* = 16) supportive, 49% (*n* = 19) unsure and 10% (*n* = 4) not supportive. Men involved in workshops (*n* = 28) identified informational needs to assist in treatment decision‐making and side‐effect management as the top priority throughout care. Men described support groups and practice nurses as key enablers. Short consultation times and complex information were described as barriers. Unlike HPs, *all* men supported the inclusion of a PRO comparator tool in a portal.

**Conclusions:**

Our findings suggest that a patient support portal should provide information in lay terms that address the shifting priorities of men with PCa. Men with PCa would welcome the development of a portal to centralize support information and a PRO comparator tool to prompt health‐seeking behaviour. Future research will implement these findings in the development of a portal, and pilot and evaluate the portal within a population‐based sample.

**Patient or Public Contribution:**

This project adopted a codesign approach including both men with PCa and HPs involved in PCa care. Men with PCa also formed part of the study's steering committee and consumer advisory groups. HPs were consulted in a serious of online focus groups. Subsequently, men with PCa and their support persons participated in workshops. Men with PCa were also involved in the preparation of this manuscript.

## 1 INTRODUCTION

Prostate cancer (PCa) is the second most common cancer in men worldwide, with an estimated 1.1 million new cases diagnosed in 2012.^1^ The 5‐year relative survival rate in Victoria in 2014–2018 was 92%.^2^ Yet, many men live with symptoms that affect their urinary, sexual and bowel function, impacting their quality of life (QoL).^3^

In 2009, the Prostate Cancer Outcomes Registry of Victoria (PCOR‐Vic) was established to monitor variation in patterns of presentation and care. In addition to collecting clinical information, the registry collects patient‐reported outcomes (PROs) using the Expanded Prostate Cancer Index Composite (EPIC‐26).^4^ This information is fed back to hospitals and clinicians to prompt action to address identified QoL issues and improve service delivery, but is not currently returned to men who complete the EPIC‐26. An efficient mechanism to improve QoL among men with PCa at a population level may be to use PCOR‐Vic to provide a personalized web interaction and allow self‐management. Due to its wide coverage, PCOR‐Vic can provide men with individual feedback, acting as a unique instrument for contact with men with PCa.

Patient portals provide an innovative opportunity to engage men in self‐managing their disease by integrating PROs to prompt health‐seeking behaviour; yet, little evidence exists about their utility in supporting men with PCa. A large UK population‐based study of 35,000 men with PCa assessed functional outcomes (using the EPIC‐26) and general health‐related QoL 18–42 months after diagnosis.^5^ This study informed the development of the ‘Men like Me’ portal, enabling men to see the outcomes of other men like them using the data from the study (controlled for age, stage of disease and treatment type). However, an evaluation of the portal is yet to be published. The ‘Men like Me’ portal used human‐centred design to develop a personalized PRO dashboard through a series of iterative focus groups with men with PCA, HPs and design experts.^6^ These focus groups provided guidance for optimizing the design of the PRO dashboard^6^ as a means to providing feedback to impacted men. As such, developing a portal that provides information and resources customized to the needs of Australian men, building on the ‘Men like Me’ portal, offers the potential to support Australian men with PCa.

The aim of this qualitative action research study was to identify and integrate the perspective of healthcare professionals (HPs), men and their support persons on:

1. priority issues facing men with PCa,
2. barriers and enablers to accessing care and support information,
3. the acceptability of a PRO comparator tool and
4. the format, language and organization of information on a web portal (‘BroSupPORT’).

## 2 MATERIALS AND METHODS

**Figure 1:**
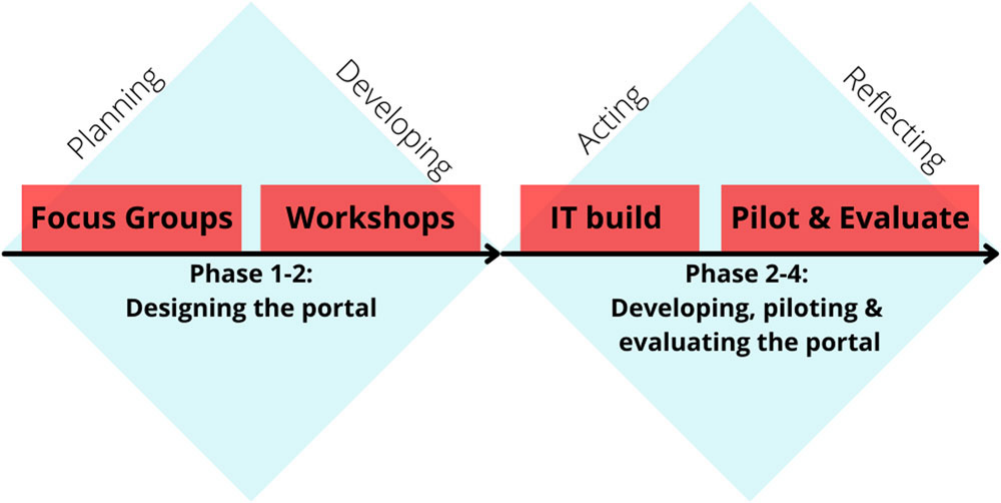
The action research process informing the development of BroSupPORT

This study adopted a qualitative action research process to develop the BroSupPORT portal. Action research consists of four main phases—planning, developing, acting and reflecting.^7^ In this paper, we report on the first two phases of the action research process, which incorporates work related to both the focus groups and workshops, as shown in Figure 1. Phases 3–4 of the study will be outlined in a subsequent paper.

### 2.1 Overall governance

A steering committee was established comprising representatives from the Department of Health and Human Services Victoria, Alfred Health, Monash University, Movember, the Urological Society of Australia and New Zealand and the Victorian Agency of Health Information and two men with PCa. The Committee was chaired by a representative of the Project Sponsor. The core research team met with the consumer, research and clinical advisory groups before the focus groups and before each workshop to receive feedback on data collection processes, recruitment and research findings.

### 2.2 Focus groups

#### 2.2.1 Participants

Purposive sampling was used to recruit HPs treating, managing or supporting a man with PCa from four hospitals contributing to the PCOR‐Vic and the Prostate Cancer Foundation Australia (PCFA). Given that focus groups were to be conducted online, it was agreed that the optimal sampling size of each focus group would be no more than 10, to allow the opportunity for active engagement and visibility of participants. A list of specialities including urologists, oncologists, nurses, physiotherapists, psychologists, general practitioners (GPs), radiotherapists and exercise physiologists was provided to the site principal investigator and they were asked to invite participation among these groups at urology multidisciplinary team meetings (MDMs) and via direct email to those specialties not attending MDMs. A snowballing approach was used to recruit other specialty group members.

#### 2.2.2 Data collection

Due to restrictions enforced by the COVID‐19 pandemic, focus groups were held online using Zoom Video Communications^8^ and MURAL.^9^ Clinicians unable to attend a focus group were invited to participate in an interview. Focus groups were conducted with HPs with the aim of exploring issues that men with PCa face following treatment, the top priorities for men posttreatment, recommendations for men based on the top priorities, the barriers and enablers to accessing supportive care and the inclusion of a comparator tool for men to compare their PROs with other men at a similar age, stage of disease and treatment type using data from the relevant clinical quality registry, in this case, PCOR‐Vic.

#### 2.2.3 Analysis

Thematic analysis^10^ was undertaken to categorize deidentified qualitative data contained within transcripts. Themes were selectively coded by iteratively cross‐checking each transcript to ensure significant representation of a major theme. Deductive and inductive coding was used, with data analysed using NVivo 12.^10^

### 2.3 Codesign workshops

#### 2.3.1 Participants

Men from PCOR‐Vic who had previously completed a PRO survey via email (approximately 12 months after diagnosis) were invited to participate. Men were emailed an explanatory statement via PCOR‐Vic and an advertisement was posted on the Cancer Council Victoria (CCV) website. In addition, men participating in the PCFA Pathfinder Registry (a register for men willing to participate in research) and engaged in PCFA support groups were forwarded the advertisement. We screened men expressing interest on application to ensure that they had access to the internet at home, could speak English and whether their support person would be interested in participating in one workshop. Purposive sampling of those who expressed interest was performed to suit a range of treatment modalities, age groups, demographic profile and online workshop size. Men were informed that they would be reimbursed with a $50 gift card for each workshop that they attended to compensate for their time. Eligible men completed a consent form electronically. Men were sent activities to prepare before Workshop 1.

#### 2.3.2 Data collection

Workshop content was developed iteratively by the research team, with input from Consumer, IT and Research Advisory groups. The research team reviewed workshop objectives and desired outcomes before developing a draft topic guide and slides. The Consumer Advisory Group met before each workshop to ensure that (1) content was in lay language and (2) workshop activities were suitable for men who may not be experienced with technology, and realistic timing was allocated to activities. The Research Advisory Group met before Workshop 3 to explore how to present a PRO comparator tool feature to men with PCa, as well as to incorporate the perspectives of support persons.

In total, there were seven workshops, with preworkshop and workshop activities described in Table 1. Focus Group findings were used to guide workshop content development. Workshop 1 was conducted in two sessions (Workshop 1a and Workshop 1b) to cater for likely attrition, and Workshop 3 was conducted in two sessions (one with and the other without support person). Data were collected in a mixture of formats both during and after workshops: Google Docs, Qualtrics survey and printed activities were used. Two scribes took field notes at each workshop. Field notes were reviewed by the core project team weekly and used to develop content for subsequent workshops.

**Table 1:** Themes, aims and respective activities for each workshop

Workshop no.	Theme	Aim	Activity	Activity description
Preworkshop 1		To obtain demographic details from participants	Survey	Complete a data collection form capturing demographic information—age, marital status, highest level of education and health insurance type
1	Exploring men's PCa care journey	Understand men's PCa journey and the issues that they faced	River of Life (preworkshop)	Participants were asked to illustrate their PCa journey graphically
		Explore priority issues for men and how portal content can be developed to address them	Problem Trees (preworkshop)	Participants were asked to think of a tree as a problem that they experienced, with the branches representing the effects and the roots representing the cause of the problem
2	Understanding how men addressed issues that they faced	Identify barriers and enablers to accessing support	Mind Map of Issues (during‐workshop)	A mind map developed using Fitch's Supportive Care domains to conceptualize and organize issues raised through their River of Life and Problem Trees
		Develop a list of resources that men used to address the issues that they faced	Priorities survey (postworkshop)	Survey developed using Qualtrics that asked participants to rate on a scale of 1–10 how much a priority an issue was during either diagnosis and treatment or during recovery and long‐term care
3	Incorporating partners and spouses	Understand whether men wanted to compare their outcomes with other men at a similar age, stage of disease and treatment type	Vote & discussion (during workshop)	Initial voting poll and subsequent discussion on the inclusion of a comparator tool, concerns and support
		Incorporate the perspectives of partners and spouses on which resources where available to men and how we can encourage health‐seeking behaviour	Recommendations Table (during workshop + postworkshop)	An excel spreadsheet that displayed the priorities ranked from highest to lowest (average score) and the corresponding recommendations or resources that men used to address them
4	Incorporating the perspectives of health professionals	To introduce recommendations of health professionals and to decide upon the format of information	Presenting data in different formats. Reviewing and endorsing focus group recommendations	Discussion of voting on what format to present information and education, and sharing men's stories (video, written, graphics, online communities, etc.)
				Sought feedback and endorsement on recommendations that health professionals recommended as helpful that had not been raised by consumers yet
5	Organizing information into the site map	Finalize key messaging and develop a site map	Messaging for consent and comparison information and organizing information into a site map	Sought feedback on messaging and description required for when men consent to access the portal and before the comparison information is revealed. Facilitators used an online interactive whiteboard (Mural) to refine and create umbrella terms for organizing information in the portal, and sorting what topics and information go under each term

Abbreviation: PCa, prostate cancer.

### 2.4 Analysis

A range of analysis techniques were used to suit the unique activities and methods of each workshop. We arranged issues raised in Workshop 1 into categories described in Fitch's Supportive Care Framework^11^ using MURAL, and descriptive‐content analysis^12^ was undertaken. Descriptive statistics were used to present frequencies, range and mean scores of each priority in Workshop 2. In Workshop 3, participants were given the opportunity to discuss the strategies that they had used to address priority issues that helped or hindered their recovery, as well as what additional information they would have liked in relation to each priority area. Zoom polling allowed participants to vote on the acceptability of a PRO comparator tool (Workshop 3). In Workshop 4, men were asked to comment on the format of information that they preferred (1) when hearing stories from other men and (2) when receiving information and educational material. In Workshop 5, the ‘consent and warning message’ for the portal was sent to participants for feedback ahead of the workshop. We compiled a master version of notes from the workshop discussion and additional email feedback received after the relevant workshop. Key headings of the portal and how the priority information would be organized were determined by consensus during the workshop.

This study was approved by Alfred Health Ethics Committee (59472, Project 714/9) and Monash University HREC (24688). Site‐specific governance approval was obtained from each organization from which focus group members were recruited.

## 3 RESULTS

### 3.1 Phase 1: Planning

The focus group participant characteristics are described in Table 2. Thirty‐nine HPs attended focus groups, and two were interviewed. Most (41%) were allied HPs, followed by medical professionals (36%) and hospital‐based nurses (13%). Most HPs were recruited from public hospitals and worked in the metropolitan area.

**Table 2:** Details of focus group participants

**Group**	**Specialty**	***N*/total**
Health professional group		
Medical		
	Urologist	4/39
	Palliative care specialist	1/39
	General practitioner	1/39
	Medical oncologist	3/39
Nurse		
	Hospital‐based nurses	5/39
	PCFA nurses	4/39
Allied Health		
	Physiotherapist	4/39
	Psychologist/psycho‐oncologist	3/39
	Exercise physiologist	3/39
	Radiation therapist	2/39
	Dietician	2/39
	Sexual therapist	1/39
	Social worker	1/39
Location of work for the focus group member		
Metropolitan hospital		22/39
Regional hospital		17/39
Employment service type		
Private hospital		10/39
Public hospital		18/39
Private practice		1/39
University		1/39

Abbreviation: PCFA, Prostate Cancer Foundation Australia.

#### 3.1.1 Focus group key findings

Seven key themes emerged from HP focus groups regarding important issues to consider when developing an information portal for men. Quotes corresponding to specific themes are presented in Table 3.

**Table 3:** Details of focus group participants

Focus group theme	Quote	Health professional
Shifting priorities for men	‘Priorities change throughout the whole continuum from the time of diagnosis to acute treatment to survivorship stage’	GP, metro, private
	‘They shift because they get on top of other concerns, so if their urinary continence was their big concern, if they get that under control then they might worry about their sexual function’	Nurse, metro, private
Information needs	‘Patients need information and they need it at their level’	Urologist, metro, public
	‘We gave them written information and you know this great booklet … they got to page 5, and what they really wanted is a DVD, so we made a DVD of the booklet, they seemed to like that more…’	Radiotherapist, metro, public
Multidisciplinary nature of managing the disease	‘…highlighting [to men] the multidisciplinary nature of how care can be delivered and referring to other professionals with experience dealing with these kinds of issues…making sure men can get the best quality of care’	Sexual therapist, metro, private
Importance of support groups and connection	‘…[support groups are] life changing for a lot of men’	Exercise physiologist, metro, university
	‘…there are a lot of gentlemen that come in who say I've had a friend who has had brachytherapy or I've had a friend who has had this, so I think this [hearing from other men] would be a good thing to include’	Palliative care specialist, metro, private
Importance of partners and support persons	‘I think with relationships sometimes you find the partner wants to tell you more than the actual patient of what's really happening at home so you know you get a lot of information out of them’	Social worker, metro, public
Social determinants of health impacted access to care	‘…Injections cost a lot of money, they're not covered through the PBS (Pharmaceutical Benefits Scheme). Even for a vacuum erection device. Private health insurance may provide a rebate for some of it, a public patient will have to pay for the thing outright. The costs of treatment for… erectile dysfunction management are significant’	Nurse, metro
	‘I've had a patient in for counselling in relation to erectile dysfunction who needed an interpreter, but when the interpreter arrived and was female… that was a cultural issue for the man’	Nurse, regional
	‘They're pretty reluctant to talk about it [erectile dysfunction] or elaborate on it or pretty reluctant to get more information on – they just seem a bit embarrassed and not wanting that information from their physio at that point in time, just that [lack of] openness to talking about it and seeking treatment is what I've experienced’	Physiotherapist, metro, private
	‘Numeric rating scales are very good way yes? So, I mean the DT (Distress Thermometer) is a thermometer to measure distress but if you are measuring basic symptoms just draw a ruler 0‐10 and 0 is no symptom and 10 is very severe symptom… Sometimes we see them with faces underneath, really happy smiley face and miserable face at the end’	Palliative care specialist, metro

Abbreviation: GP, general practitioner.

##### Shifting priorities for men

In the immediate 12‐month period following treatment, HPs reported that men are initially concerned with dealing with the immediate side effects of treatment, such as urinary issues and sexual function, but that these change over time.

##### Information needs

HPs highlighted the importance of providing men with relevant and accessible information to inform treatment decision‐making as well as side‐effect management. This also included the format of the information, with several participants highlighting that men preferred video content as opposed to written information.

##### Multidisciplinary nature of managing the disease

To address the range of treatment side effects experienced by men during the survivorship stage of their PCa, several participants underlined the importance of a multidisciplinary approach to care. Providing access to cancer‐specific professionals was seen as critical to addressing the wide variety of issues that men face after treatment and was highlighted as an important inclusion in the portal.

##### Importance of support groups and connection

Across all focus groups and interviews, HPs signified the importance of men engaging with other men with PCa. Hearing from other men with PCa raises awareness of services or pathways with which men might not be familiar. HPs highlighted that often, men found it comforting to speak to other men with PCa. Support groups were commonly highlighted as providing men with opportunities to engage with other men with PCa and raising awareness of services such as cancer gyms, which provide a physical space for men to exercise as well as network with other men and HPs. HPs endorsed including stories from other men as a key feature in a portal.

##### Importance of partners and support persons

Partners were highlighted as playing a critical role in supporting men with PCa. Specifically, participants emphasized how partners often feel compelled to raise issues with HPs that men did not. Some HPs noted that without partners accompanying men in consultations, men would seldom engage in discussion. Conversely, some participants stated that partners attending consultations could act as a barrier for men in discussing sensitive topics with their HP.

##### Social determinants of health impacted access to care

Costs of treatment and ongoing costs were identified by HPs as causing an added burden and distress for some men, particularly in relation to use of sexual aids and medication to manage side effects of treatment. Several HPs raised the importance of considering the cultural needs of men when accessing information and care. Participants noted that some men preferred to speak to another male practitioner and with an HP who had a similar cultural background. HPs working in regional areas highlighted the difficulty that men living in rural, regional and remote areas face in accessing support services, particularly PCa‐specific specialists such as pelvic floor physiotherapists and continence nurses. Several HPs raised the challenge of raising sensitive and often stigmatized topics such as erectile dysfunction in the confines of a short consultation. Many HPs highlighted disparities in accessing supportive care services for men within public and private health systems, particularly in relation to the reduced capacity to access a practice nurse in the public health services. Practice nurses were particularly important in providing information to men at their level, assisting them in managing expectations and sourcing continence products and other aids.

HPs reported that many men had poor health and technology literacy and that this posed a significant barrier to them accessing online support services. HPs highlighted the spectrum of patients’ health literacy levels, with one participant providing men with the latest research articles while, in contrast, others used simple figures and diagrams to assist in communicating complex anatomical information or treatment options. Resources written in lay language were regarded as useful tools to assist men with low health literacy. Disparities in health and technology levels, as well as access to the internet, were magnified in regional, rural and remote areas.

##### HPs conflicted on the usefulness of the comparator tool

There were mixed views on the inclusion of a PRO comparator tool in the portal by HPs, with some reporting that it might alleviate feelings of isolation and provide men with assurance by comparing their outcomes against others who had received the same treatment and were of the same age and stage of disease. However, some HPs highlighted that the PRO comparator tool might create unnecessary distress among men whose outcomes were substantially worse than those of other men, that it might be difficult for men to interpret the graphs and terminology (e.g., normal vs. not normal or expected vs. not expected) and that they would be unable to support men in the aftermath of seeing the report. HPs working in regional settings expressed more concerned with including a PRO comparator tool than those in metropolitan settings.

#### 3.1.2 Consumer workshop key findings

Most men involved in codesign workshops were between 70 and 80 years old (51%), diagnosed with intermediate‐risk disease (43%), received surgery as their primary PCa treatment (71%), were married (57%) and listed a bachelor's degree or higher as their highest level of education (Table 3). Participant retention was defined as attending three of the five workshops. Sixty‐one percent of men attended three of the five workshops (Table 4).

**Table 4:** Characteristics of the consumers who participated in the codesign workshops

Characteristic	Workshop 1A (*n* = 15)	Workshop 1B (*n* = 13)	Workshop 2 (*n* = 15)	Workshop 3A (*n* = 12)	Workshop 3B (*n* = 6 + 5 partners)	Workshop 4 (*n* = 15)	Workshop 5 (*n* = 17)
Age							
<60	2	2	2	1	1	2	2
60‐70	4	5	5	3	1	4	4
70‐80	8	6	7	8	3	9	10
≥80	1	0	1	0	1	0	0
Diagnosing NCCN risk group^a^							
Low	3	1	2	1	1	2	2
Intermediate	5	2	4	3	0	2	2
High	4	1	3	4	1	5	4
Very high	0	1	0	0	0	0	0
Metastatic	1	0	0	1	0	0	1
Treatment type							
Surgery	9	11	13	9	5	11	13
External beam radiation	3	0	1	3	0	2	0
Brachytherapy	2	1	1	0	1	1	1
ADT	4	0	1	3	0	1	3
WWAS	1	0	1	0	1	0	1
Marital status^b^							
Married (and not separated)	8	8	10	6	5	10	10
Widowed	1	1	1	2	0	1	2
Separated	0	0	0	0	0	0	0
Divorced	3	0	2	2	1	3	3
Single	0	1	1	1	0	1	1
Highest level of education^b^							
Tertiary—bachelor degree or higher	6	4	6	6	1	5	6
Advanced diploma or diploma	3	3	5	2	4	6	6
Certificate Level III or IV	1	3	3	2	1	3	3
Year 12 or equivalent	1	0	0	0	0	0	0
Year 10–11	0	0	0	0	0	0	0
Year 9 or less	0	0	0	1	0	0	0
Private health insurance							
Yes	6	5	7	4	5	8	9
No	6	5	7	7	1	7	7

Abbreviations: ADT, androgen deprivation therapy; NCCN, national comprehensive cancer network; WWAS, watchful waiting or active surveillance.

^a^ Some data are not available as clinical knowledge is required, and could not be collected from the participants.

^b^ Data may be incomplete due to data collection forms that were not returned.

#### 3.1.3 Workshop 1: Understanding men's PCa journey

A wide range of issues faced by men with PCa were raised through the River of Life activity (Multimedia Appendix S1) and the subsequent Problem Tree activity (Multimedia Appendix S2). Men highlighted that initially, they faced many physical side effects, such as urinary incontinence and leakage, erectile dysfunction, hot flushes and weight gain. Men also raised practical issues such as caring for others and financial distress; emotional issues such as anxiety, withdrawal and fear of recurrence; and psychological issues such as loss of libido, impact on masculinity and confidence. Informational needs were frequently referenced to support treatment decision‐making and reduce uncertainty by setting expectations.

#### 3.1.4 Workshop 2: Exploring priority issues

A mind map was developed by the project team after Workshop 1 using Fitch's Supportive Care Needs Framework^10^ and discussed during Workshop 2 (Multimedia Appendix S3). Men stated that a survey should be distributed after Workshop 2 to rank the 56 issues that men identified across (1) the early stage of their PCa journey (diagnosis through to and including treatment) and (2) the recovery and longer‐term period. In total, 23 men (82%) completed the survey after Workshop 2.

#### 3.1.5 Workshop 3: Barriers and enablers to address priority issues and views on the comparator tool

Workshop 3 discussed the survey results, focussing on the top 10 issues that men identified in the early stage and the recovery/long‐term period, shown in Table 5. During diagnosis and treatment, participants described the importance of having access to support groups and hearing stories from other men to understand different treatment options, the expected recovery outcomes and potential side effects. Men and their support persons highlighted that speaking to their urologist or GP, accessing online resources such as the PCFA website, CCV or through videos of other men documenting their treatment journeys on YouTube helped them to obtain relevant information in the early stage of their journey. Most notably, workshop participants described the overwhelming pressure placed on them to make a treatment decision and feeling overwhelmed by the enormity of the decision. Short consultation times with specialists and difficulty finding information in lay language were identified as key barriers to addressing informational needs.

**Table 5:** Top 10 priority issues during diagnosis and treatment and during recovery and long‐term care identified in Workshop 3

Rank	Priority/issue	Mean
During diagnosis and treatment		
1	Informational domain: Understanding the extent of diagnosis	8.6
2	Informational domain: Understanding and monitoring PSA	8.6
3	Informational domain: Finding relevant information	8.5
4	Informational domain: Making a treatment decision	8.4
5	Practical domain: My doctor being able to answer my questions	8.1
6	Psychological domain: Dealing with cancer and moving on	7.8
7	Physical domain: Urinary issues (including incontinence)	7.4
8	Physical domain: Being able to get an erection	6.6
9	Social domain: How treatment would affect my relationship with my partner	6.4
10	Informational domain: Being able to understand what aids and tools (such as pumps, tablets and injections) can help me to have an erection	6.2
During recovery and long‐term care		
1	Informational domain: Understanding and monitoring PSA	8.2
2	Informational domain: Understanding the chances of recurrence	8.2
3	Physical domain: Recovery	8.2
4	Informational domain: Understanding prognosis	7.8
5	Physical domain: Physical activity	7.7
6	Practical domain: Monitoring PSA	7.7
7	Informational domain: Finding relevant information	7.6
8	Physical domain: Urinary issues (i.e., incontinence)	7.0
9	Physical domain: Being able to get an erection	6.8
10	Social domain: Impact on sex life	6.3

Abbreviation: PSA, prostate‐specific antigen.

During recovery and long‐term care, some men and their support persons again highlighted the importance of support groups in establishing expectations and normalizing symptoms that they may still be feeling. Men also highlighted the lifestyle changes that they had made to their diet and exercise, which assisted in recovery. Men and partners were asked to vote anonymously on inclusion of the PRO comparator tool on the portal, with all participants favouring its inclusion, stating that it would provide men with a sense of reassurance and encourage men to be proactive in their self‐management. Participants noted that information and resources should be supplemented with information on support services, especially if they became distressed or upset by the tool. Men stressed the importance of presenting the information in the comparator tool in lay language and for the comparator tool to be optional, with a warning displayed to men before they proceed to view their comparison. Participants were provided with three options of how the comparator tool might display their information (Multimedia Appendices [Link], [Link], [Link]) as a scale, gauge chart or bar chart. Men preferred the bar chart, whilst a preference for the scale chart emerged in the workshop for men and their support person.

#### 3.1.6 Workshop 4: Presenting data and examining barriers and enablers provided by HPs

**Figure 2:**
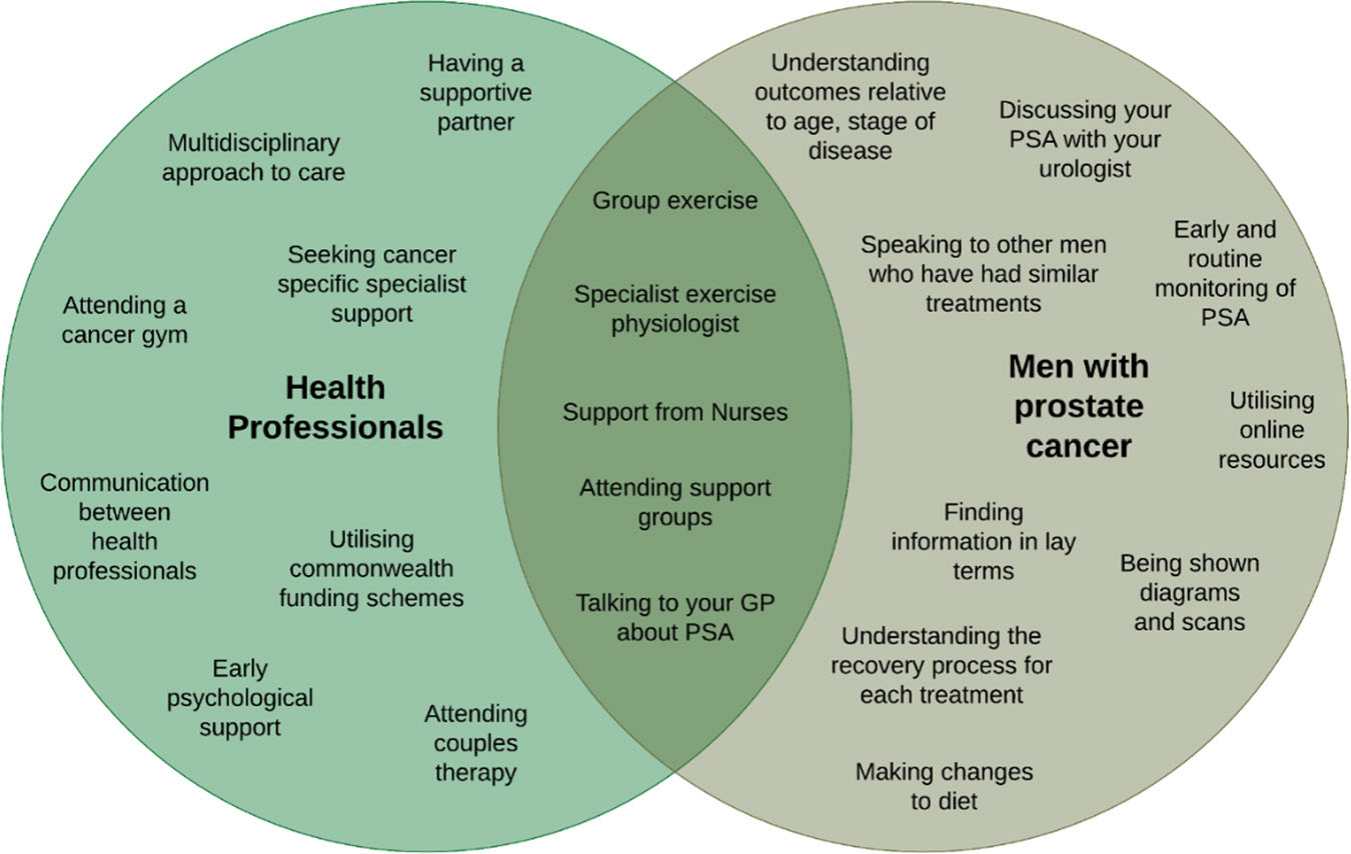
Comparison of the enablers to receiving high‐quality prostate cancer care from the perspectives of health professionals and men with PCa discussed in Workshop 4. PCa, prostate cancer

**Figure 3:**
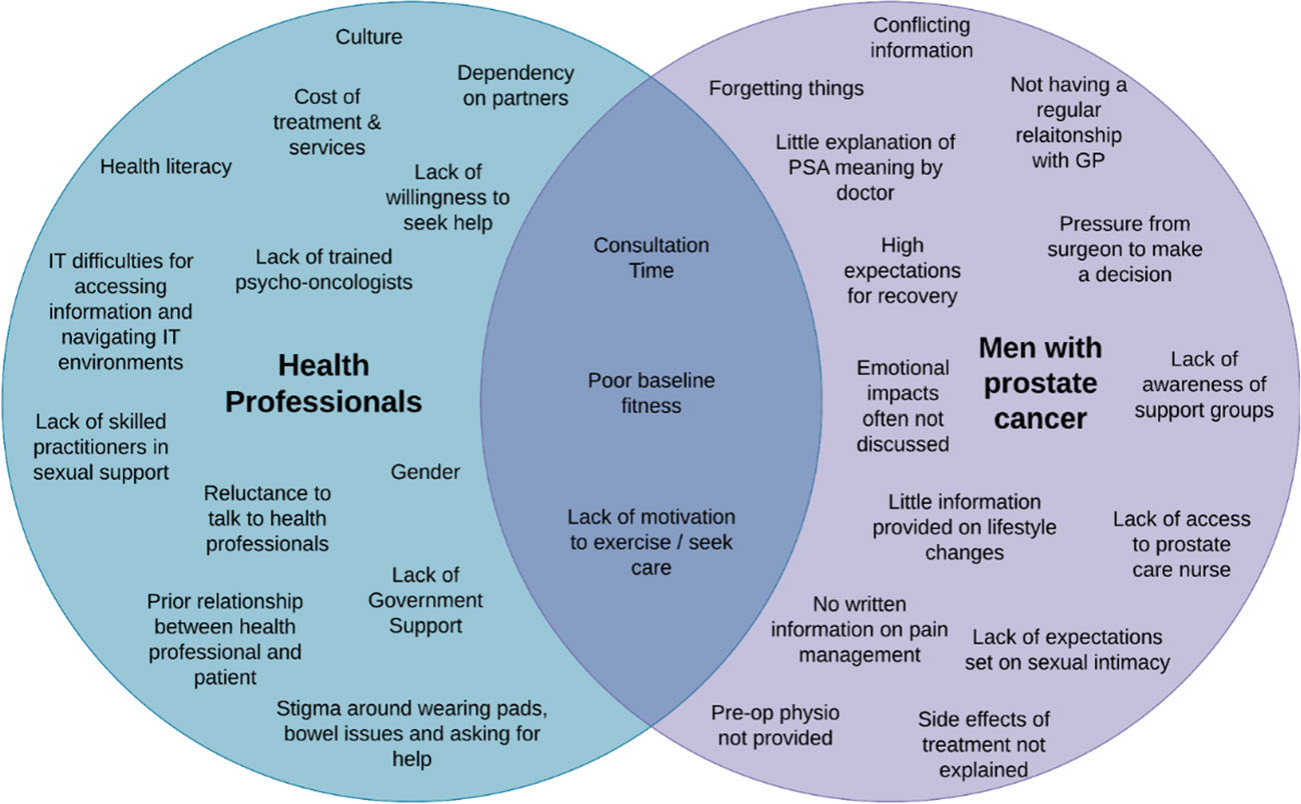
Comparison of the barriers to receiving high‐quality prostate cancer care from the perspectives of health professionals and men with PCa discussed in Workshop 4. PCa, prostate cancer

Workshop 4 focused on determining how to best present data and explored issues raised by HPs in focus groups. Most workshop participants preferred that men's stories be delivered as video content (67%). Yet, some men (20%) stated that they would prefer to read men's stories in a short, written format using clear and lay language. There was no clear preference for presenting education and information. Men wanted to see use of a variety of formats, infographics, figures and statistics, video content and fact sheets, incorporated into the portal. Suggested video content included PCFA webinars and other short videos from YouTube. A directory with details on what HPs recommended for PCA‐specific issues, such as how to find local HPs, and referral paths, was reported as a valuable resource. Men reviewed priority issues identified by HPs in focus groups to include in the BroSupPORT web portal and agreed with all suggestions, other than not including information on mindfulness for stress. Figures 2 and 3 compare the enablers and barriers identified by HPs and men.

#### 3.1.7 Workshop 5: Messaging of informed consent and content layout in the portal

Workshop 5 presented men with a proposed approach to accessing the portal after men are recruited to the PCOR‐Vic and have completed a QoL survey online.^13^ It was proposed that after completion of the survey online, men would receive an electronic consent form to complete before entering the BroSupPORT web portal. Participants stated that the consent page should be brief, with the option for men to view the entire privacy statement and document if they choose. A short description of BroSupPORT should mention that the portal was designed with and for men with PCa. Men emphasized that they should be given the choice of whether they want to ‘reveal’ the comparison information or skip it ahead to the supportive information. Messaging before the comparator is revealed should include a clear description of what the tool will present, explain the number of other individuals the comparison is based on, a note that the tool includes sensitive information and indication of support services should the comparator information cause them distress.

Participants were provided with a list of strategies identified in Workshop 3 to address the top 10 priority issues and a summary of enablers and barriers to receiving high‐quality care (Figures 2 and 3). They were asked to consider ways of organizing the information and the categories under which information could be organized. Participants agreed on six different headings to help men easily navigate the portal and find information. Following this, participants in the workshop collectively used the Q‐Sort methodology to place each issue under a respective category.^14^ The sorting exercise enabled the group to reach consensus on the headings and content that will belong under each category. This process produced a proposed site map for the BroSupPORT web portal as shown in Multimedia Appendix S7.

## 4 DISCUSSION

The objective of this study was to determine priority issues facing men with PCa, barriers and enablers to accessing care, format and organization of information on a portal and whether HPs and men with PCa would support the inclusion of a PRO comparator tool within the portal. We used a variety of data collection approaches including focus groups, interviews and codesign workshops. Focus group findings were used to inform workshop activities and content. Barriers, enablers and recommendations to accessing high‐quality PCa care were captured during both focus groups and workshops and were compared. While there was some overlap, an important finding of this study was that there were many unique views not shared between the two groups. The synthesis of this study resulted in a template design for a web portal by men.

Barriers and enablers to accessing supportive care have been widely cited in the literature by men with PCa, their partners, HPs and other care providers.^15^, ^16^, ^17^, ^18^, ^19^, ^20^, ^21^ Studies have suggested that men may be reluctant to seek help from HPs because it is perceived as a threat to their social and personal identity^21^ and that this reluctance increases with age.^22^ In contrast, study participants were forthcoming with their experiences in workshops, perhaps reflecting greater acceptance of their diagnosis, that the large proportion of men in our study had a spouse and that men were well educated.^23^ HPs and men with PCa highlighted the pivotal role that partners and support groups played in providing support. Support groups enable men to engage in their health with other similar men.^18^, ^24^ HPs and men with PCa in our study identified barriers to accessing supportive care to address side effects of treatment. Urinary incontinence,^17^, ^18^ erectile dysfunction,^19^, ^21^ fatigue,^16^, ^17^, ^18^, ^20^, ^21^ lack of motivation^16^, ^17^, ^18^ and stigmas associated with wearing pads^19^, ^20^, ^21^ were highlighted in our study as treatment side effects, and have been well documented in the literature. These side effects adversely impact men's ability to return to work and engage in relationships with others.^19^, ^20^

A promising finding of our study is that a substantial number of men with PCa already use online support resources and would welcome a portal to centralize support information to prompt health‐seeking behaviour.

Our finding that informational needs are a high priority throughout care for men with PCa is concordant with the literature.^15^, ^25^, ^26^, ^27^, ^28^, ^29^, ^30^, ^31^, ^32^ Studies have shown that a lack of information may lead to uncertainty, anxiety and distress in some men.^30^ Furthermore, men with PCa have reported lack of clarity about the arrangements for treatment and types of treatment available, lack of information about the practicalities of managing incontinence, on local support groups and on how to effectively self‐manage side effects and improve physical recovery.^5^, ^33^ Some studies have highlighted that out of all unmet needs of men with PCa, informational needs are the most important.^25^ However, informational needs are often identified through online or postal surveys, with low response rates^25^, ^34^ and nonrepresentative cohort studies.^25^, ^30^, ^31^ Studies capturing unmet needs are often cross‐sectional in nature, which renders them unable to capture men's needs over time.^25^, ^30^, ^32^, ^34^, ^35^ Our study highlights the importance of a web portal providing information to cater to men at different stages of their PCa care and presented in a variety of formats.

Little is known about the acceptability of PRO integration into a portal^6^ and there is no literature on how the portal might address supportive care needs once comparisons are made using a PRO comparator tool. Our finding that men and partners support the inclusion of a PRO comparator tool may reflect an increasing desire to empower consumers with information to help them take control of their disease.^36^ This relates to a fundamental principle of medical ethics, that of patient autonomy, an important ethical consideration for healthcare providers.^37^ A PRO comparator tool has the potential to support men who may be reluctant to disclose embarrassing symptoms to their clinicians but may feel compelled to understand how to manage their side effects or learn more about their outcomes from the privacy of their own home. This is seen in the few studies in the literature that highlight that men with PCa want to be able to compare their PRO data with other similar men as well as personalize the type of comparison that they see.^6^, ^38^ Access to personalized information through an online portal enables patients to better prepare for their consultations, which in turn improves clinician–patient communication, a critical component of patient‐centred care.^39^ As such, this tool aims to promote health‐seeking behaviour amongst men with PCa.

While overwhelmingly patients support inclusion of a comparator tool, this was not supported by all. The literature to date has identified HPs concerns regarding patients accessing test results via a patient portal,^39^, ^40^ but few studies examine the perspectives of clinicians regarding men comparing their PROs with other similar men.^6^, ^38^ A systematic review has identified a volume–outcome relationship, where health services performing low rates of PCa surgery had worse outcomes compared to high‐volume services.^41^ Concerns of HPs in this study included the potential for causing distress or poor presentation of the information. These concerns, mainly raised by regional HPs, may be attributed to anxiety around patients having poorer outcomes compared to their metropolitan counterparts. HPs practising in regional and rural areas may be reluctant for men to compare their outcomes to men who have been treated in metropolitan areas because of fear of not performing well, even if this is unfounded. As we only conducted focus groups in one regional health area, it is unclear whether these findings can be generalized to HPs from other regional areas. Further research is required to evaluate the risks associated with providing men with the ability to compare the PRO outcomes with other men through a patient portal.

This is the first study to use a codesign approach to inform the content of a patient support portal for men with PCa. Building on design principles used to develop the ‘Men like Me’ portal,^5^ our study used codesign principles to incorporate, compare and contrast the perspectives of both HPs and men with PCa on how supportive care needs can be addressed through an online portal. By incorporating the views of HPs, men and support providers, the portal is arguably more likely to be supported and recommended by HPs to their patients. Through the support of HPs, a portal may help reduce the survivorship burden and gap in support provision for men with PCa. Online conduct of focus groups and workshops may have increased attendance and the diversity of participants, reducing geographical restrictions for participants, especially due to the extraordinary circumstances presented during the pandemic. While the methods used in this study had to pivot from patient‐facing to online, online workshops may have been more conducive for some men to share their experiences and issues with the research team in comparison to a face‐to‐face workshop due to the degree of anonymity that online workshops afford.^15^

Yet, despite these strengths, there were also important limitations, particularly in relation to the bias of the included cohort in the project. Men in our study were, on average, older and had a reported a higher rate of surgery as their primary treatment compared to the Victorian population of men contributing to the PCOR‐Vic.^42^ Men who could not speak English were excluded from the study and we did not specifically undertake to understand views of men according to their sexual orientation and, as such, cannot comment on whether these factors impact the priorities, barriers and enablers and views of the comparator tool. Workshops were held during business hours, meaning that working men could not keep attending workshops. Workshop participants were well educated, may have been more invested in their health, had more time to volunteer or may have displayed better knowledge of supportive care services than those who did not respond to the study invitation. Ultimately, these biases may impact the generalizability of our findings, and further investigation of these populations is warranted.

## 5 CONCLUSION

Designing platforms that encourage patient self‐management is important, considering the fragmentation of supportive care delivery for men with PCa.^15^, ^43^ This study outlines men's preferences regarding the content and format of information displayed on an online PCa support portal. The next phase of this study will involve evaluating the comparator tool and support information within the portal with a wider range of men. If the portal is deemed acceptable by men with PCa, further investigation may include capture of PROs and presentation of a comparison in real time.

## CONFLICT OF INTERESTS

The authors declare that there are no conflict of interests.

## AUTHOR CONTRIBUTIONS

Susan Evans, Jeremy Millar and Ellie Tsiamis designed the research project. Benjamin Shemesh, Ellie Tsiamis, Darshini Ayton and Jacinta Opie carried out the research. Prassanah Satasivan, Paula Wilton, Karla Gough, Katrina Lewis, Colin O'Brien, Max Shub, Amanda Pomery and Christopher Mac Manus were involved in planning and supervizing the research. Benjamin Shemesh performed the analysis, drafted the manuscript and designed the figures. All members were involved in editing the manuscript.

## Supporting information

Supporting information.

Supporting information.

Supporting information.

Supporting information.

Supporting information.

Supporting information.

Supporting information.

## Data Availability

The data that support the findings of this study are available on request from the corresponding author, Jeremy Millar and Susan Evans. The data are not publicly available due to restrictions, for example, their containing information that could compromise the privacy of research participants.
