# Supplementary figures and images for: Codesigning a patient support portal with health professionals and men with prostate cancer: An action research study
Source: Health Expect. 2022 Apr 11;25(4):1319–31. doi: 10.1111/hex.13444 (PMC9327875; doi:10.1111/hex.13444)

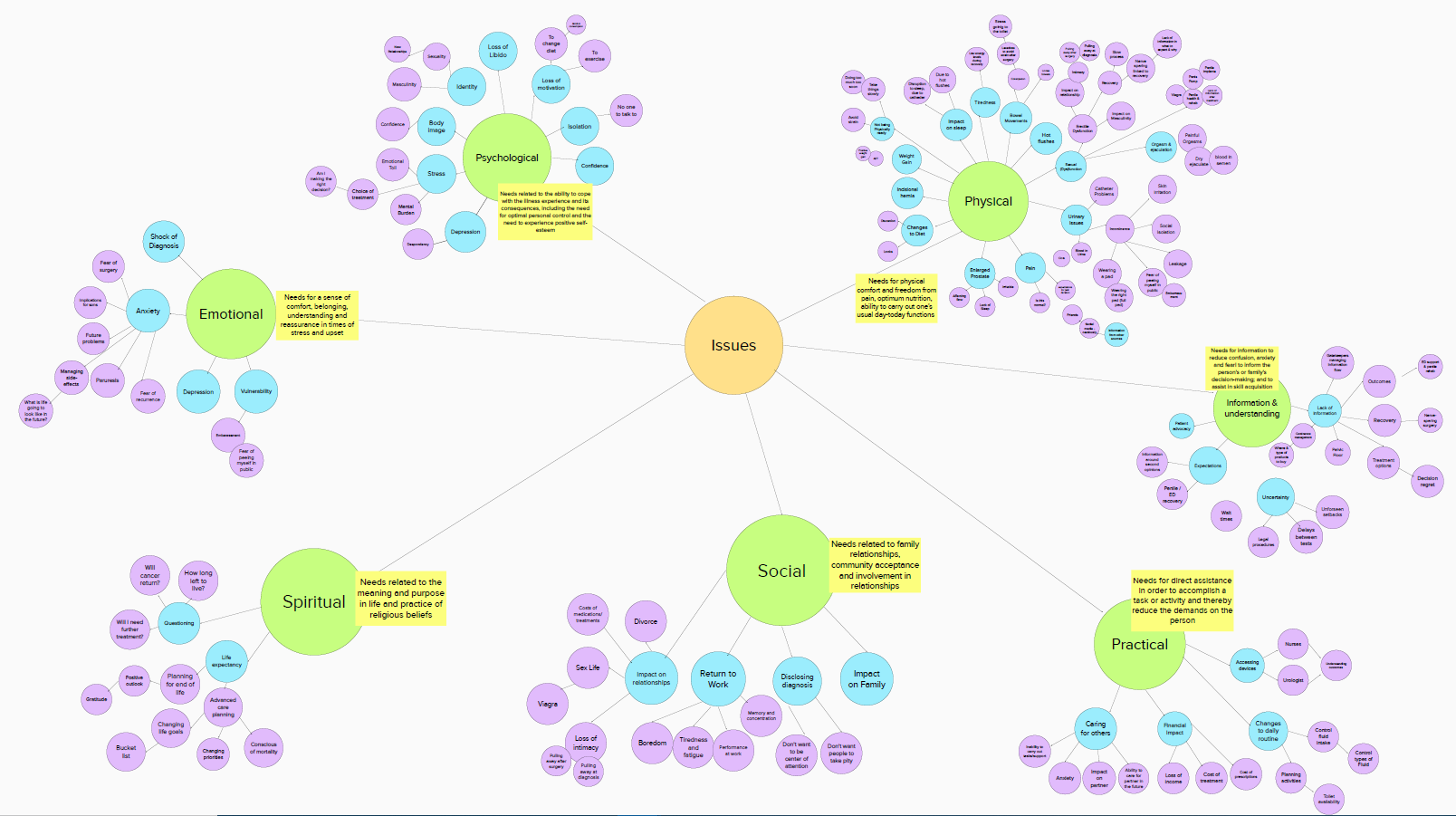

Supplement: Supplementary file 1 — Supporting information. [file HEX-25--s007.png]

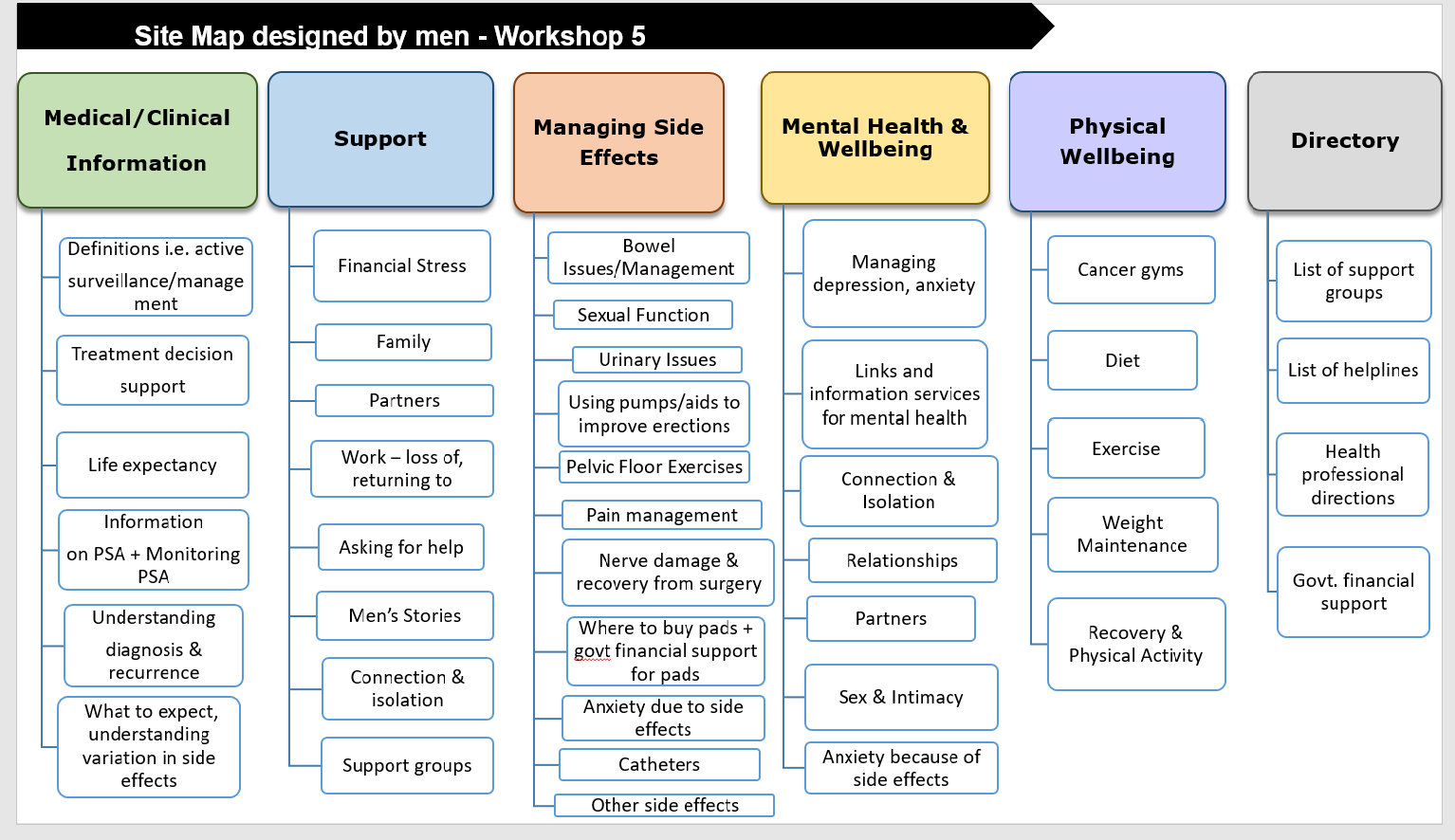

Supplement: Supplementary file 2 — Supporting information. [file HEX-25--s002.png]

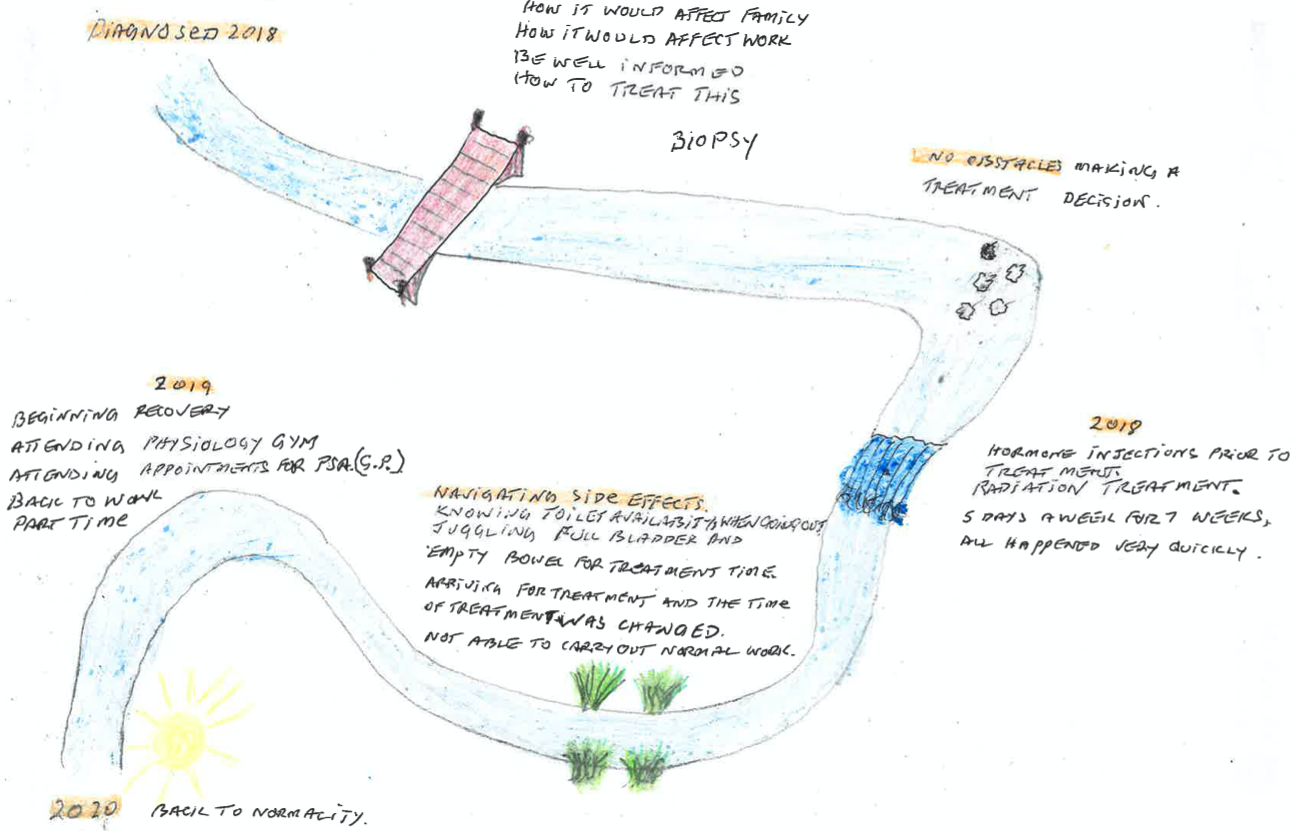

Supplement: Supplementary file 3 — Supporting information. [file HEX-25--s001.png]

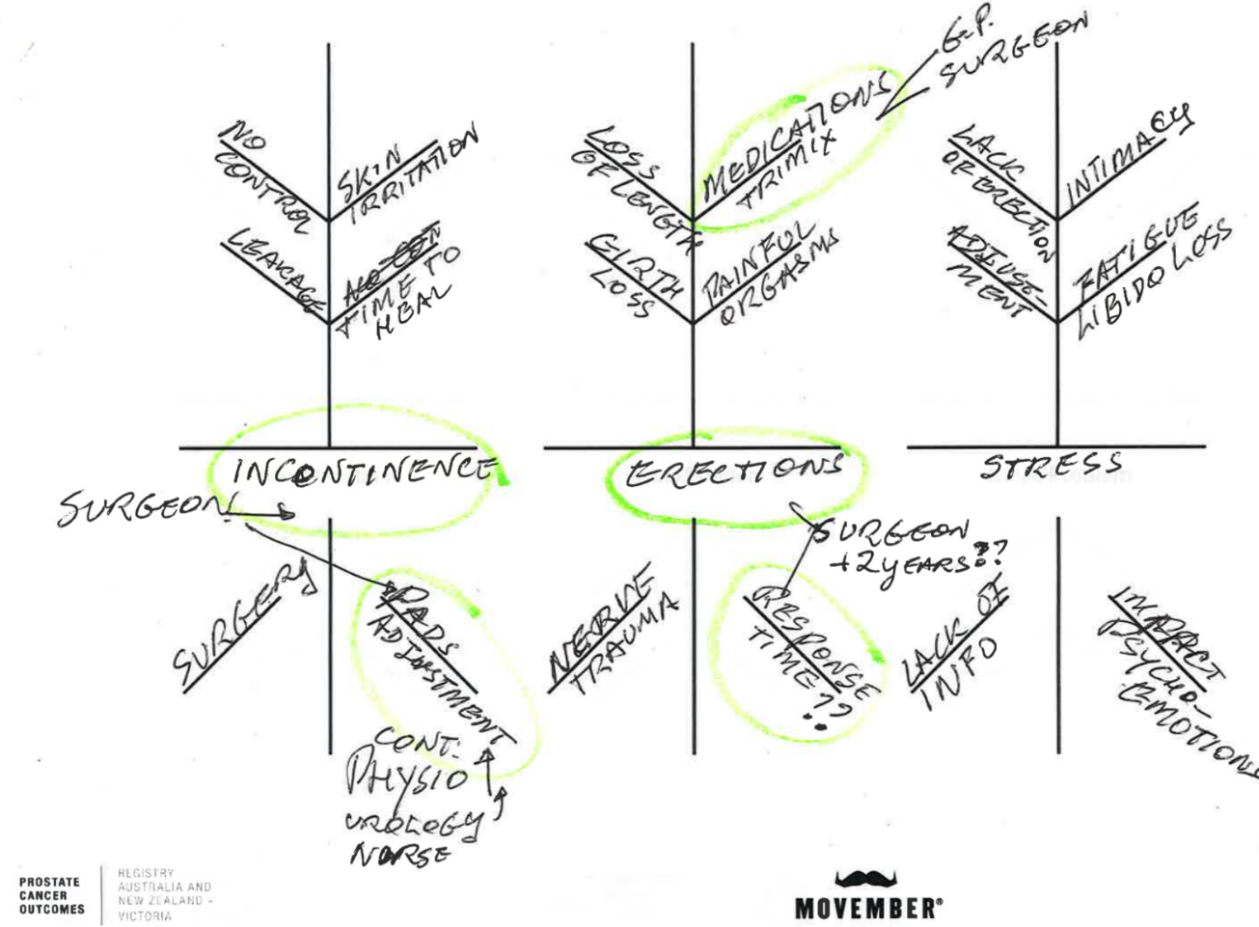

Supplement: Supplementary file 4 — Supporting information. [file HEX-25--s006.png]

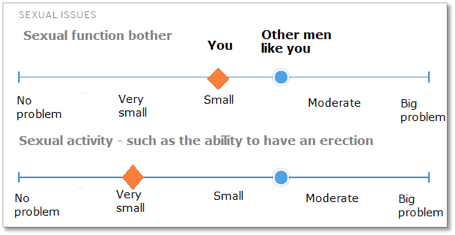

Supplement: Supplementary file 5 — Supporting information. [file HEX-25--s004.png]

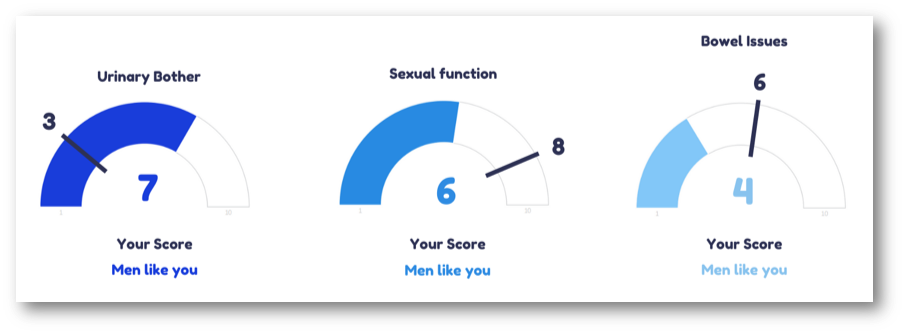

Supplement: Supplementary file 6 — Supporting information. [file HEX-25--s003.png]

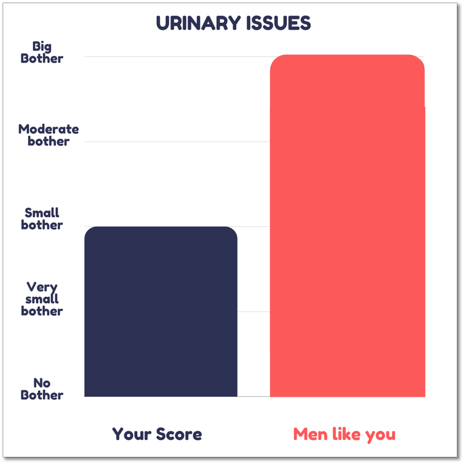

Supplement: Supplementary file 7 — Supporting information. [file HEX-25--s005.png]
